# Trends in remission rates for rheumatoid arthritis in England and Wales: a population-level cohort study

**DOI:** 10.1093/rheumatology/keaf233

**Published:** 2025-05-05

**Authors:** Edward Alveyn, Callum Coalwood, Gráinne Farrell, Ella Jackson, Maryam A Adas, Sam Norton, Peter Lanyon, Elizabeth Price, Joanna M Ledingham, James B Galloway, Mark D Russell

**Affiliations:** Centre for Rheumatic Diseases, King’s College London, London, UK; Department of Rheumatology, King's College Hospital NHS Foundation Trust, London, UK; British Society for Rheumatology, London, UK; British Society for Rheumatology, London, UK; British Society for Rheumatology, London, UK; Centre for Rheumatic Diseases, King’s College London, London, UK; Department of Rheumatology, King's College Hospital NHS Foundation Trust, London, UK; Centre for Rheumatic Diseases, King’s College London, London, UK; Institute of Psychiatry, Psychology & Neuroscience, King's College London, London, UK; Department of Rheumatology, Nottingham University Hospitals NHS Trust, Nottingham, UK; Lifespan and Population Health, School of Medicine, University of Nottingham, Nottingham, UK; Department of Rheumatology, Great Western Hospital NHS Foundation Trust, Swindon, UK; Rheumatology Department, Portsmouth Hospitals University NHS Trust, Portsmouth, UK; Centre for Rheumatic Diseases, King’s College London, London, UK; Department of Rheumatology, King's College Hospital NHS Foundation Trust, London, UK; Centre for Rheumatic Diseases, King’s College London, London, UK; Department of Rheumatology, King's College Hospital NHS Foundation Trust, London, UK

**Keywords:** RA, remission, window of opportunity, time-to-treatment, care quality, epidemiology

## Abstract

**Objectives:**

Considerable data support early treatment of RA to obtain disease remission. Data from the National Early Inflammatory Arthritis Audit (NEIAA) in England and Wales suggest that, despite recent improvements in referral-to-treatment times, remission rates remain unchanged. We investigated reasons for this disconnect by evaluating temporal trends, geographical variation and predictors of remission in individuals with new RA diagnoses.

**Methods:**

An observational cohort study of individuals with RA was conducted using data from NEIAA (May 2018–April 2024). Temporal and geographical variation in remission rates (DAS28 < 2.6) were explored using interrupted time-series and case-mix-adjusted mixed-effects regression. Predictors of remission were assessed using multivariable logistic regression.

**Results:**

13 752 of 21 904 (62.8%) individuals with RA had data on DAS28 at 3 months after initial rheumatology assessment, of whom 4764 (34.6%) achieved remission. National remission rates were stable from 2018 to 2024; however, wide geographical variation was observed, ranging from 28.4% (London) to 40.3% (East of England). Threefold differences in remission rates were seen between individual hospitals within regions. Younger age, female sex, Black ethnicity, higher baseline DAS28, delayed DMARD initiation and longer symptom duration were independently associated with reduced odds of remission. Delays between symptom onset and referral have increased since the COVID-19 pandemic.

**Conclusion:**

While national remission rates for early RA have remained stable in England and Wales since 2018, there is marked regional and hospital-level variation, highlighting ongoing inequities in service delivery. Addressing factors beyond referral-to-treatment time—particularly delayed presentation to primary care—is required to improve remission rates.

Rheumatology key messages

- Remission rates for RA vary markedly between regions and hospitals in England and Wales.
- Age, sex, ethnicity, baseline DAS28 and time-to-treatment are independent predictors of early remission.
- Delays between symptom onset and referral to rheumatology have increased since the COVID-19 pandemic.

## Introduction

Numerous studies have demonstrated that early initiation of disease-modifying anti-rheumatic drugs (DMARDs), such as methotrexate, increases the likelihood of remission in individuals with RA [1]. Remission is associated with improved outcomes for people living with RA, including prevention of joint damage, disability and comorbidities (e.g. cardiovascular disease), as well as economic benefits [2]. As such, remission is the primary therapeutic goal for many individuals with early RA [3, 4].

The UK National Institute for Health and Care Excellence (NICE) recommends that DMARDs should be commenced within 6 weeks of referral to rheumatology services for new diagnoses of RA, followed by monthly monitoring, with the aim of maximizing the likelihood of achieving remission [3, 5]. The National Early Inflammatory Arthritis Audit (NEIAA) was established in 2018 to monitor performance against these NICE Quality Standards throughout England and Wales [6]. Reports are produced on an annual basis, outlining national, regional and hospital-level performance in metrics including referral-to-treatment times and the proportion of patients achieving remission within 3 months of initial rheumatology assessment.

The sixth NEIAA annual report was published in October 2024 [7]. This report showed an 8% improvement in the proportion of patients commencing DMARDs within 6 weeks of referral, from 52% in the year to April 2023% to 60% in the year to April 2024. Despite this improvement, and considerable variability in this metric since 2018, the proportion of patients achieving remission within 3 months of initial assessment has remained static, at ∼35% [7]. We sought to investigate the potential reasons for this disconnect between referral-to-treatment times and remission rates using data from NEIAA. Our specific aims were to evaluate temporal and geographical variation in remission rates and explore factors associated with the attainment of remission in individuals with new diagnoses of RA in England and Wales.

## Methods

### Data source and study sample

An observational cohort study was performed using data from NEIAA, a national audit programme that monitors the quality of care provided to people with inflammatory rheumatological diagnoses in the National Health Service (NHS) of England and Wales [6]. Since 8 May 2018, it has been mandatory for NHS providers of rheumatology services in England and Wales to submit data to NEIAA on patients, aged 16 years or over, newly referred with suspected early inflammatory arthritis diagnoses [6]. The sample for this study was all people enrolled in NEIAA between 8 May 2018 and 30 April 2024 who had diagnoses of RA (determined on the basis of clinical judgement by the treating clinician) and who were eligible for follow-up under an early inflammatory arthritis pathway [6].

### Outcome measures

The primary outcome of interest was the attainment of remission (defined as a disease activity score in 28 joints [DAS28] <2.6) at the 3-month follow-up visit after the initial rheumatology assessment. Where both DAS28-ESR and DAS28-CRP were available for individuals, data on DAS28-ESR were utilized in the primary analyses. As sensitivity analyses, results were presented using DAS28-CRP in preference over ESR, and using the Boolean definition of remission (tender joint count, swollen joint count, patient global assessment [0–10 scale] and CRP [mg/dl] all ≤1) [8].

### Temporal analyses

Baseline sociodemographic, disease, assessment and treatment-related characteristics were presented descriptively for individuals with RA, separated by year of initial rheumatology assessment and region of England and Wales. Temporal changes in the proportion of individuals achieving remission at 3 months were displayed graphically using two-way line plots. Additional plots were used to describe temporal trends in the proportion of individuals who: (i) were in low (DAS28 2.6–3.1), moderate (DAS28 3.2–5.1) or high (DAS28 > 5.1) disease activity states at 3 months; (ii) had good, moderate or no DAS28 response at 3 months, relative to baseline (as defined by the European Alliance of Associations for Rheumatology [EULAR] [9]); (iii) achieved remission by 12 months after initial rheumatology assessment (these data stopped being collected in NEIAA after April 2023); (iv) were initiated on DMARDs within 6 weeks of referral to rheumatology services (a quality metric recommended by NICE [5]); (v) were initiated on DMARDs within 12 weeks of referral to rheumatology services; (vi) had symptom durations of 6 months or greater prior to being referred to rheumatology services; (vii) were referred to rheumatology services within 3 days of presenting to their primary care clinician with symptoms suggestive of early inflammatory arthritis (a quality metric recommended by NICE [5]). Where indicated, moving-average smoothing was applied to temporal trends, whereby each data point represents the average of the current month, the two preceding months and the two subsequent months. Interrupted time-series analyses (ITSAs) were used to explore temporal trends in outcomes before and during the COVID-19 pandemic. Single-group ITSA was employed, with Newey-West standard errors and lags used to account for autocorrelation between observation periods [10].

### Predictors of remission

Logistic regression was used to explore factors associated with achieving remission at 3 months. Variables were selected *a priori* from those collected in NEIAA on the basis of whether they were felt to be potentially important predictors of remission, as follows: age, sex (recorded by clinicians from patients’ medical records), ethnicity (categorized in NEIAA as White, Black, Asian, mixed or other ethnicity), disease severity at initial assessment (DAS28), autoantibody status (RF and/or CCP positive), the duration of symptoms prior to referral to rheumatology (collected in NEIAA as a categorical variable: 0–3 months, 3–6 months, 6–12 months, >1 year), time from receipt of referral to initiation of DMARDs (categorized as: 0–3 weeks, 3–6 weeks, 6–12 weeks, >12 weeks), the initiation of a methotrexate-based treatment regimen within 3 months of initial assessment and whether corticosteroids were prescribed at initial assessment. No data on the dose or duration of methotrexate or corticosteroid therapy are collected in NEIAA. Estimates from multivariable models incorporating all covariates were presented as adjusted odds ratios (aORs) with 95% CIs. Adjusted marginal probabilities of achieving remission were presented where indicated, alongside estimates from univariable and age- and sex-adjusted models. Nesting of patients within hospitals was accounted for using cluster-robust standard errors. Variables with missing data were imputed using multiple imputation with chained equations (20 imputed datasets), with Rubin’s rules used to combine the datasets. Logistic regression was used to impute binary variables (autoantibody status), multinomial logistic regression was used to impute unordered categorical variables (ethnicity), ordinal logistic regression was used to impute ordered categorical variables (symptom duration; referral-to-treatment time) and linear regression was used to impute continuous variables (baseline DAS28). Models were restricted to individuals with data available on the primary outcome (remission at 3 months). Included predictors with complete data were age, sex and hospital.

As sensitivity analyses, generalized ordinal logistic regression was used to explore the association between symptom duration prior to referral, referral-to-treatment time and the odds of achieving at least one category improvement in DAS28 by 3 months after the initial rheumatology assessment. In these models, estimates represented the odds of achieving a less active DAS28 category (high, moderate, low disease activity or remission) than the baseline category, to those equally or more active than the baseline category. Proportional odds assumptions were met for symptom duration and referral-to-treatment times, and all estimates were adjusted for age, sex, ethnicity, autoantibody status, DAS28 at baseline, methotrexate initiation by 3 months and corticosteroid use at baseline, relaxing proportional odds assumptions.

### Regional and hospital-level variation

Geographical variation in 3-month remission rates was presented by region (defined in NEIAA as the seven NHS England commissioning regions and Wales [6]) and by treating Hospital Trust (an organizational unit of one or more hospitals serving a local population in England; in Wales, these organizational units are represented in NEIAA as Local Health Boards). The observed probabilities of achieving remission by region were presented alongside case-mix-adjusted empirical Bayes probability estimates from logistic mixed-effects regression models. Hospital Trust and region were included as random intercepts in three-level models, with patient-level factors held constant across regions. Regional variation was depicted graphically using geographical heat maps, and presented alongside the probability of achieving remission in individual Hospital Trusts within each region (estimated using empirical Bayes means).

All statistical analyses were conducted in Stata version 18 (StataCorp LLC, College Station, TX, USA). No correction was made for multiple hypothesis testing.

### Ethical approval

Approval to conduct this research using NEIAA was obtained from the Healthcare Quality Improvement Partnership (HQIP). No further ethical approval was required.

### Role of the funding source

No funding bodies had any role in study design, data collection, analysis or interpretation, manuscript writing, or in the decision to submit the article for publication.

## Results

Between 1 May 2018 and 30 April 2024, 21 904 individuals with new diagnoses of RA were enrolled in NEIAA. The mean age at diagnosis was 59.4 years (S.D., 15.5 years); 63.6% were female; 73.5% were RF and/or CCP positive and the median DAS28 at baseline was 5.0 (interquartile range, 4.0–5.9) (Table 1). 13 752 (62.8%) individuals had data available on disease activity status at 3 months after the initial rheumatology assessment, of whom 4764 (34.6%) achieved remission (DAS28 < 2.6).

**Table 1.** Characteristics of individuals with RA diagnoses enrolled in NEIAA, separated by year of initial assessment (12 month periods, starting in May of each year)

	Total	2018/19	2019/20	2020/21	2021/22	2022/23	2023/24
	*N* = 21 904	*N* = 5153	*N* = 3907	*N* = 1438	*N* = 3735	*N* = 3820	*N* = 3851
Mean age, years [S.D.]	59.4 (15.5)	59.5 (15.1)	59.5 (15.5)	58.1 (16.0)	59.7 (15.7)	59.2 (15.6)	59.4 (15.5)
Sex							
Male	7977 (36.4%)	1858 (36.1%)	1471 (37.7%)	514 (35.7%)	1349 (36.1%)	1397 (36.6%)	1388 (36.0%)
Female	13 927 (63.6%)	3295 (63.9%)	2436 (62.3%)	924 (64.3%)	2386 (63.9%)	2423 (63.4%)	2463 (64.0%)
Ethnicity							
White	18 331 (84.8%)	4460 (87.1%)	3351 (86.9%)	1177 (84.6%)	3137 (84.6%)	3137 (83.0%)	3069 (81.4%)
Black	608 (2.8%)	153 (3.0%)	99 (2.6%)	33 (2.4%)	92 (2.5%)	111 (2.9%)	120 (3.2%)
Asian	1788 (8.3%)	350 (6.8%)	285 (7.4%)	114 (8.2%)	315 (8.5%)	350 (9.3%)	374 (9.9%)
Mixed/other	900 (4.2%)	158 (3.1%)	123 (3.2%)	67 (4.8%)	165 (4.4%)	181 (4.8%)	206 (5.5%)
Missing	277	32	49	47	26	41	82
RhF or CCP positive							
No	5474 (26.5%)	1396 (28.8%)	1041 (28.6%)	412 (30.4%)	939 (26.8%)	851 (23.7%)	835 (22.6%)
Yes	15 155 (73.5%)	3458 (71.2%)	2600 (71.4%)	942 (69.6%)	2565 (73.2%)	2738 (76.3%)	2852 (77.4%)
Missing	1275	299	266	84	231	231	164
Median DAS28 at baseline [IQR]	5.0 (4.0, 5.9)	5.0 (4.0, 5.9)	4.9 (3.9, 5.9)	5.0 (4.0, 5.9)	5.0 (4.0, 6.0)	5.0 (4.0, 5.9)	4.9 (3.9, 5.8)
Missing	1741	278	304	116	296	328	419
Duration of symptoms prior to referral							
<3 months	9007 (41.4%)	2171 (42.5%)	1739 (44.9%)	597 (41.9%)	1508 (40.6%)	1502 (39.6%)	1490 (38.9%)
3–6 months	5227 (24.0%)	1249 (24.4%)	924 (23.9%)	342 (24.0%)	886 (23.9%)	884 (23.3%)	942 (24.6%)
6–12 months	4045 (18.6%)	916 (17.9%)	693 (17.9%)	286 (20.1%)	690 (18.6%)	731 (19.3%)	729 (19.0%)
>1 year	3460 (15.9%)	777 (15.2%)	516 (13.3%)	199 (14.0%)	626 (16.9%)	674 (17.8%)	668 (17.4%)
Missing	165	40	35	14	25	29	22
Referral within 3 days of presentation							
No	11 166 (52.5%)	3107 (61.2%)	2174 (56.7%)	691 (49.4%)	1765 (47.7%)	1681 (45.1%)	1748 (49.2%)
Yes	10 110 (47.5%)	1966 (38.8%)	1657 (43.3%)	707 (50.6%)	1935 (52.3%)	2043 (54.9%)	1802 (50.8%)
Missing	628	80	76	40	35	96	301
Median time from referral to initial assessment, days [IQR]	17.0 (10.0, 32.0)	19.0 (11.0, 33.0)	15.0 (9.0, 28.0)	16.0 (10.0, 28.0)	18.0 (10.0, 32.0)	18.0 (11.0, 34.0)	18.0 (10.0, 33.0)
Missing	162	24	53	10	26	30	19
Time from referral to first csDMARD							
Median time, days [IQR]	36.0 (20.0, 66.0)	37.0 (21.0, 68.0)	31.0 (18.0, 57.0)	30.0 (18.0, 56.0)	39.0 (20.0, 71.0)	40.0 (21.0, 73.0)	35.0 (19.0, 63.0)
<3 weeks	5017 (26.8%)	1088 (24.2%)	991 (31.0%)	401 (31.8%)	826 (25.6%)	786 (23.6%)	925 (28.6%)
3–6 weeks	5562 (29.7%)	1363 (30.3%)	1023 (32.0%)	400 (31.7%)	881 (27.3%)	948 (28.5%)	947 (29.3%)
6–12 weeks	4927 (26.3%)	1264 (28.1%)	755 (23.6%)	274 (21.7%)	917 (28.5%)	904 (27.1%)	813 (25.2%)
>12 weeks	3237 (17.3%)	782 (17.4%)	432 (13.5%)	185 (14.7%)	599 (18.6%)	693 (20.8%)	546 (16.9%)
Missing	3161	656	706	178	512	489	620
Any csDMARD commenced by 3 months							
No	2780 (12.7%)	579 (11.2%)	618 (15.8%)	156 (10.8%)	457 (12.2%)	436 (11.4%)	534 (13.9%)
Yes	19 124 (87.3%)	4574 (88.8%)	3289 (84.2%)	1282 (89.2%)	3278 (87.8%)	3384 (88.6%)	3317 (86.1%)
Methotrexate commenced by 3 months							
No	8373 (38.2%)	1888 (36.6%)	1596 (40.8%)	568 (39.5%)	1462 (39.1%)	1389 (36.4%)	1470 (38.2%)
Yes	13 531 (61.8%)	3265 (63.4%)	2311 (59.2%)	870 (60.5%)	2273 (60.9%)	2431 (63.6%)	2381 (61.8%)
Corticosteroids prescribed at diagnosis							
No	4680 (21.8%)	1051 (20.8%)	827 (21.6%)	401 (28.4%)	776 (21.1%)	740 (19.7%)	885 (23.4%)
Yes	16 808 (78.2%)	3999 (79.2%)	2997 (78.4%)	1011 (71.6%)	2898 (78.9%)	3008 (80.3%)	2895 (76.6%)
Missing	416	103	83	26	61	72	71
DAS28 < 2.6 at 3 months							
No	8988 (65.4%)	2531 (64.0%)	1459 (64.1%)	606 (67.4%)	1585 (67.0%)	1599 (66.1%)	1208 (65.8%)
Yes	4764 (34.6%)	1426 (36.0%)	818 (35.9%)	293 (32.6%)	781 (33.0%)	819 (33.9%)	627 (34.2%)
Missing	8152	1196	1630	539	1369	1402	2016
Boolean remission at 3 months							
No	12 184 (87.1%)	3410 (86.7%)	2040 (87.3%)	799 (86.2%)	2121 (87.2%)	2176 (88.0%)	1638 (86.7%)
Yes	1807 (12.9%)	522 (13.3%)	297 (12.7%)	128 (13.8%)	311 (12.8%)	297 (12.0%)	252 (13.3%)
Missing	7913	1221	1570	511	1303	1347	1961
EULAR good response at 3 months							
No	8225 (61.7%)	2364 (61.1%)	1331 (60.8%)	541 (62.3%)	1449 (62.9%)	1456 (62.1%)	1084 (61.6%)
Yes	5108 (38.3%)	1502 (38.9%)	859 (39.2%)	327 (37.7%)	853 (37.1%)	890 (37.9%)	677 (38.4%)
Missing	8571	1287	1717	570	1433	1474	2090
Disease activity at 3 months							
DAS28 > 5.1	1759 (12.8%)	458 (11.6%)	266 (11.7%)	126 (14.0%)	344 (14.5%)	332 (13.7%)	233 (12.7%)
DAS28 3.3–5.1	4928 (35.8%)	1410 (35.6%)	808 (35.5%)	324 (36.0%)	861 (36.4%)	884 (36.6%)	641 (34.9%)
DAS28 2.6–3.2	2301 (16.7%)	663 (16.8%)	385 (16.9%)	156 (17.4%)	380 (16.1%)	383 (15.8%)	334 (18.2%)
DAS28 < 2.6	4764 (34.6%)	1426 (36.0%)	818 (35.9%)	293 (32.6%)	781 (33.0%)	819 (33.9%)	627 (34.2%)
Missing	8152	1196	1630	539	1369	1402	2016

Sociodemographic and disease characteristics are shown at baseline (the date of initial rheumatology assessment), in addition to treatment response metrics at 3 months following initial rheumatology assessment. EULAR response represents the response to treatment (e.g. csDMARDs) at 3 months following initial assessment by a rheumatologist.

Abbreviations: CCP: cyclic citrullinated peptide; csDMARD: conventional synthetic disease-modifying anti-rheumatic drug; DAS28: disease activity score at 28 joints; IQR: interquartile range.

**Figure 1.**
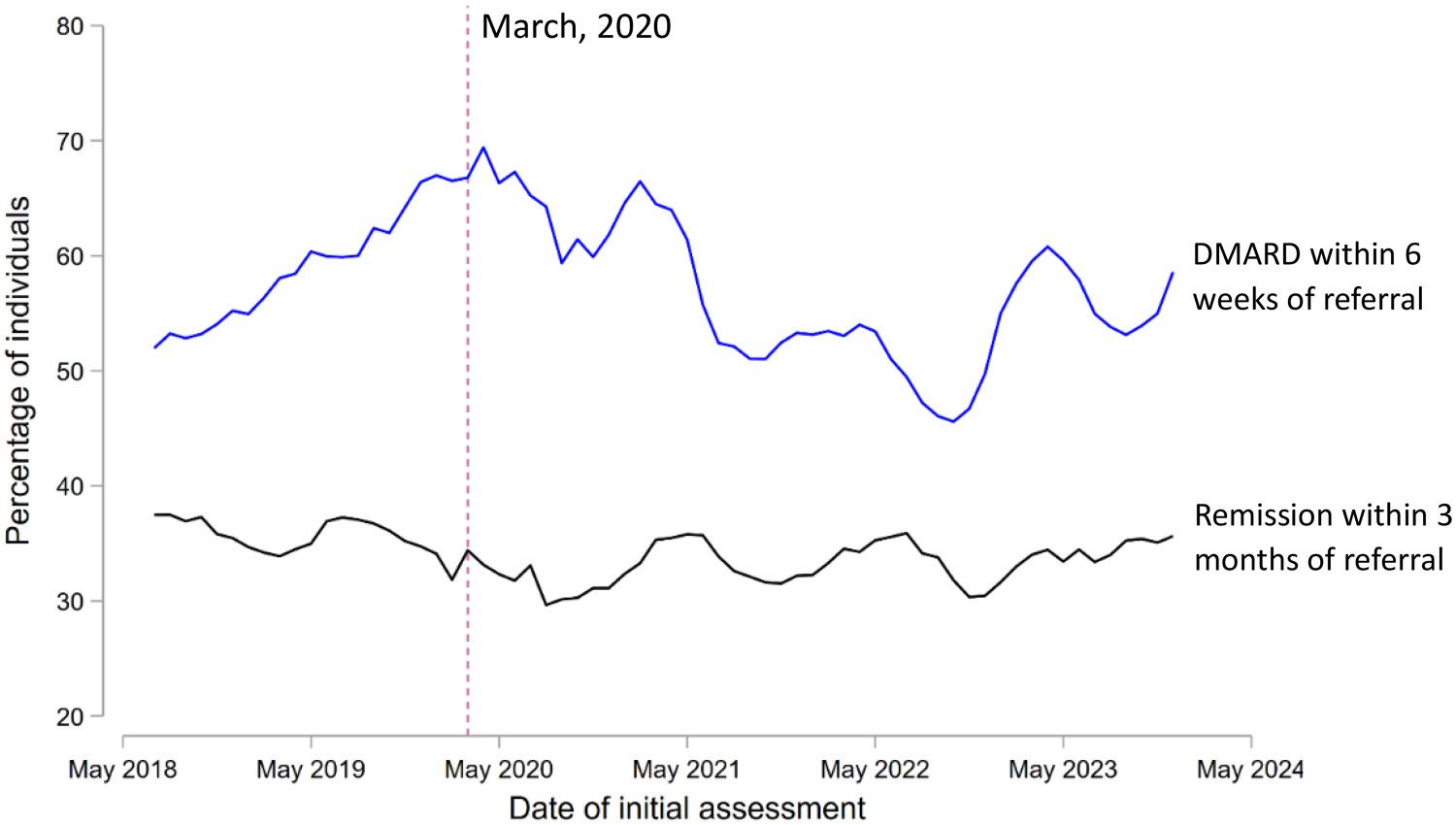
Temporal trends in the proportion of individuals with RA enrolled in NEIAA who received disease-modifying anti-rheumatic drugs (DMARDs) within 6 weeks of being referred to rheumatology services, and the proportion of all individuals with RA enrolled in NEIAA who achieved remission (DAS28 < 2.6) at the 3-month follow-up review. Smoothing has been applied, whereby each data point represents the average of the current month, the two preceding months and the two subsequent months. The vertical dashed line corresponds to the onset of the first COVID-19 pandemic lockdown in England and Wales (March 2020)

The proportion of patients achieving remission by 3 months remained broadly stable between May 2018 and April 2024, ranging from 29.7% to 37.5% (Fig. 1). Stable trends were also observed for the proportion of individuals in low, moderate or high disease activity states at 3 months, and the proportion of individuals attaining EULAR good, moderate or no treatment response at 3 months, relative to baseline (Supplementary Fig. S1, available at *Rheumatology* online). Sensitivity analyses using DAS28-CRP in preference to DAS28-ESR when both values were available showed comparable temporal trends over the study period (Supplementary Fig. S2, available at *Rheumatology* online), with 36.3% achieving remission overall. When using the Boolean definition of remission, 12.9% of individuals achieved remission at 3 months (Supplementary Fig. S2, available at *Rheumatology* online; Table 1). 5645 (25.8%) individuals had data available on disease activity states at 12 months after initial assessment, of whom 2817 (49.9%) achieved remission, with no improvement observed during the study period (Supplementary Fig. S3, available at *Rheumatology* online).

The proportion of individuals who initiated DMARDs within 6 weeks of referral to rheumatology services has changed considerably since 2018 (Fig. 1; Supplementary Fig. S4, available at *Rheumatology* online). Significant improvements in treatment times were seen in the first two years after the launch of NEIAA (9.38% improvement per year; 95% CI 7.11–11.6; *P* < 0.0001). This was followed by a sharp drop-off in performance after the onset of the COVID-19 pandemic (7.97% decrease per year; 95% CI 6.38–9.55; *P* < 0.0001), such that, by October 2022, the performance in this metric was below that at the inception of NEIAA. Since October 2022, performance has improved (8.43% improvement per year; 95% CI 0.71–16.1; *P* = 0.032); however, pre-pandemic standards of care had not yet been reached by April 2024. Overall trends were similar, but less marked, for the proportion of patients receiving DMARDs within 12 weeks of referral (Supplementary Figs S5 and S6, available at *Rheumatology* online).

In multivariable regression models, several factors were associated with the likelihood of achieving remission by 3 months (Supplementary Table S1, available at *Rheumatology* online). Older age was associated with increased odds of remission (adjusted odds ratio [aOR] per 10-year increase in age: 1.06; 95% CI 1.02–1.09; *P* = 0.0007). Women were less likely to achieve remission than men (aOR: 0.79; 95% CI 0.73–0.86; *P* < 0.0001). Individuals of Black ethnicity were less likely to achieve remission than individuals of White ethnicity (aOR: 0.67; 95% CI 0.50–0.90; *P* = 0.0071). Higher DAS28 at baseline was strongly associated with lower odds of remission (aOR per 1-unit increase in DAS28: 0.65; 95% CI 0.63–0.67; *P* < 0.0001). The probability of individuals with baseline DAS28 > 5.1 achieving remission by 3 months was 24.6%, compared with 44.0% for individuals with baseline DAS28 < 5.1 (aOR: 0.41; 95% CI 0.37–0.45; *P* < 0.0001).

**Figure 2.**
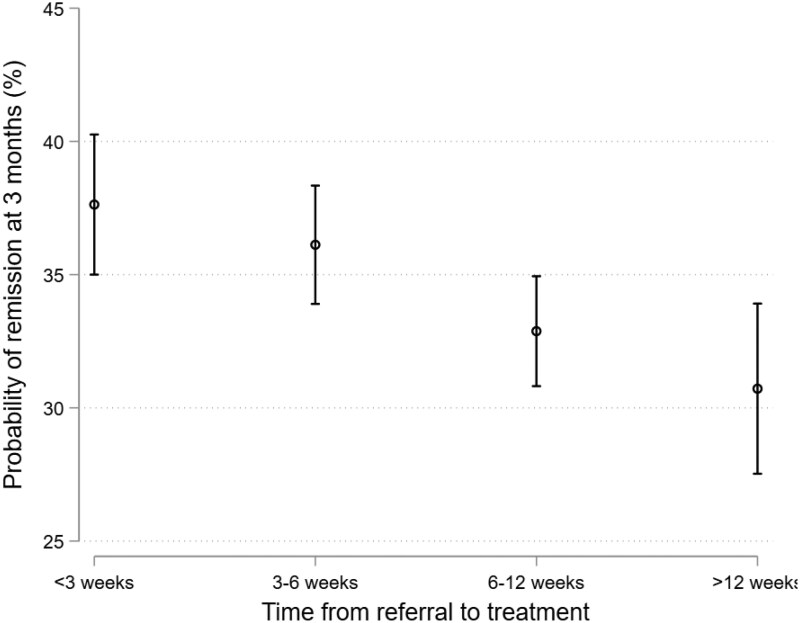
Probability of achieving remission (DAS28 < 2.6) at 3 months after initial rheumatology assessment for individuals with RA, separated by the time taken to initiate conventional synthetic DMARDs following referral to rheumatology services. Estimates are shown as marginal probabilities of achieving remission, adjusted for age, sex, ethnicity, DAS28 at baseline, autoantibody status, duration of symptoms prior to referral, initiation of a methotrexate-based treatment regimen by 3 months and corticosteroid initiation at initial assessment. Multiple imputation was used for variables with missing data

Longer delays in initiating csDMARDs after referral to rheumatology services were associated with reduced odds of achieving remission at 3 months after initial assessment, particularly for individuals waiting >12 weeks to commence treatment after referral (aOR: 0.72, 95% CI 0.60–0.86; *P* = 0.0003; Fig. 2 and Supplementary Table S1, available at *Rheumatology* online). Sensitivity analyses using generalized ordinal logistic regression to evaluate the impact of referral-to-treatment times on DAS28 activity states at 3 months showed similar findings (Supplementary Table S2, available at *Rheumatology* online), demonstrating that the observations were not confined to the remission vs. non-remission threshold but extended across other cut-offs, including shifts in high disease activity states.

**Figure 3.**
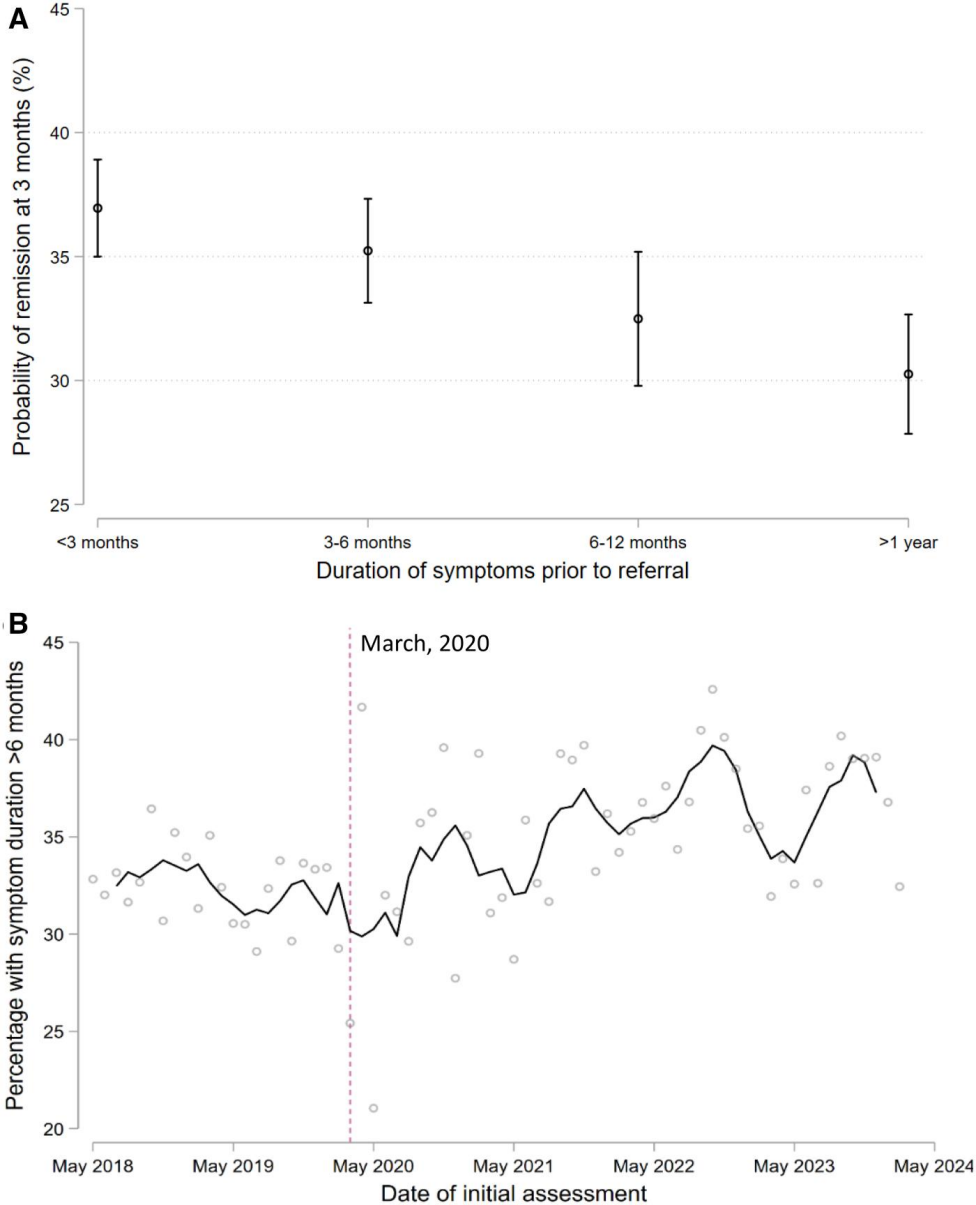
Probability of achieving remission (DAS28 < 2.6) at 3 months after initial rheumatology assessment for individuals with RA, separated by the duration of symptoms prior to referral to rheumatology services (panel A). Temporal trends in the proportion of patients reporting symptom durations of >6 months prior to referral are shown in panel B. Estimates in panel A are marginal probabilities of achieving remission, adjusted for age, sex, ethnicity, DAS28 at baseline, autoantibody status, referral-to-treatment times, initiation of a methotrexate-based treatment regimen by 3 months and corticosteroid initiation at initial assessment. Multiple imputation was used for variables with missing data. In panel B, individual data points are shown alongside a smoothed line that represents the average of the current month, the two preceding months and the two subsequent months. The vertical dashed line corresponds to the onset of the first COVID-19 pandemic lockdown in England and Wales (March 2020)

Individuals who had longer symptom durations prior to being referred to rheumatology services were significantly less likely to achieve remission at 3 months, independently of the time taken to subsequently initiate DMARDs after being referred (Fig. 3A; Supplementary Tables S1 and S2, available at *Rheumatology* online). The proportion of patients presenting with symptom durations of 6 months or more increased after the onset of the pandemic (Fig. 3B; Supplementary Fig. S7, available at *Rheumatology* online): trend prior to March 2020: 0.9% decrease per year (95% CI −1.86 to 0.0043; *P* = 0.061); trend after March 2020: 1.52% increase per year (95% CI 0.55–2.50; *P* = 0.002). In contrast, the proportion of patients referred to rheumatology services within 3 days of presenting to their primary care practitioner with symptoms suggestive of early inflammatory arthritis improved throughout the study period (Supplementary Fig. S8, available at *Rheumatology* online), indicating that the increase in symptom duration during the pandemic was due to delays that occurred prior to referral to rheumatology services.

In univariable and age- and sex-adjusted regression models, corticosteroid administration at the time of initial rheumatology assessment was associated with reduced odds of remission attainment at 3 months; however, this association was no longer present after multivariable adjustment including baseline DAS28 (aOR: 1.05; 95% CI 0.92–1.19; *P* = 0.45; Supplementary Table S1, available at *Rheumatology* online). Additionally, after multivariable adjustment, commencement of a methotrexate-based treatment regimen within 3 months of referral did not associate with early remission attainment, compared with individuals not commenced on methotrexate (aOR: 1.09; 95% CI 0.99–1.21; *P* = 0.088). Of note, however, no data were available on the dose or duration of corticosteroid or methotrexate therapy.

**Figure 4.**
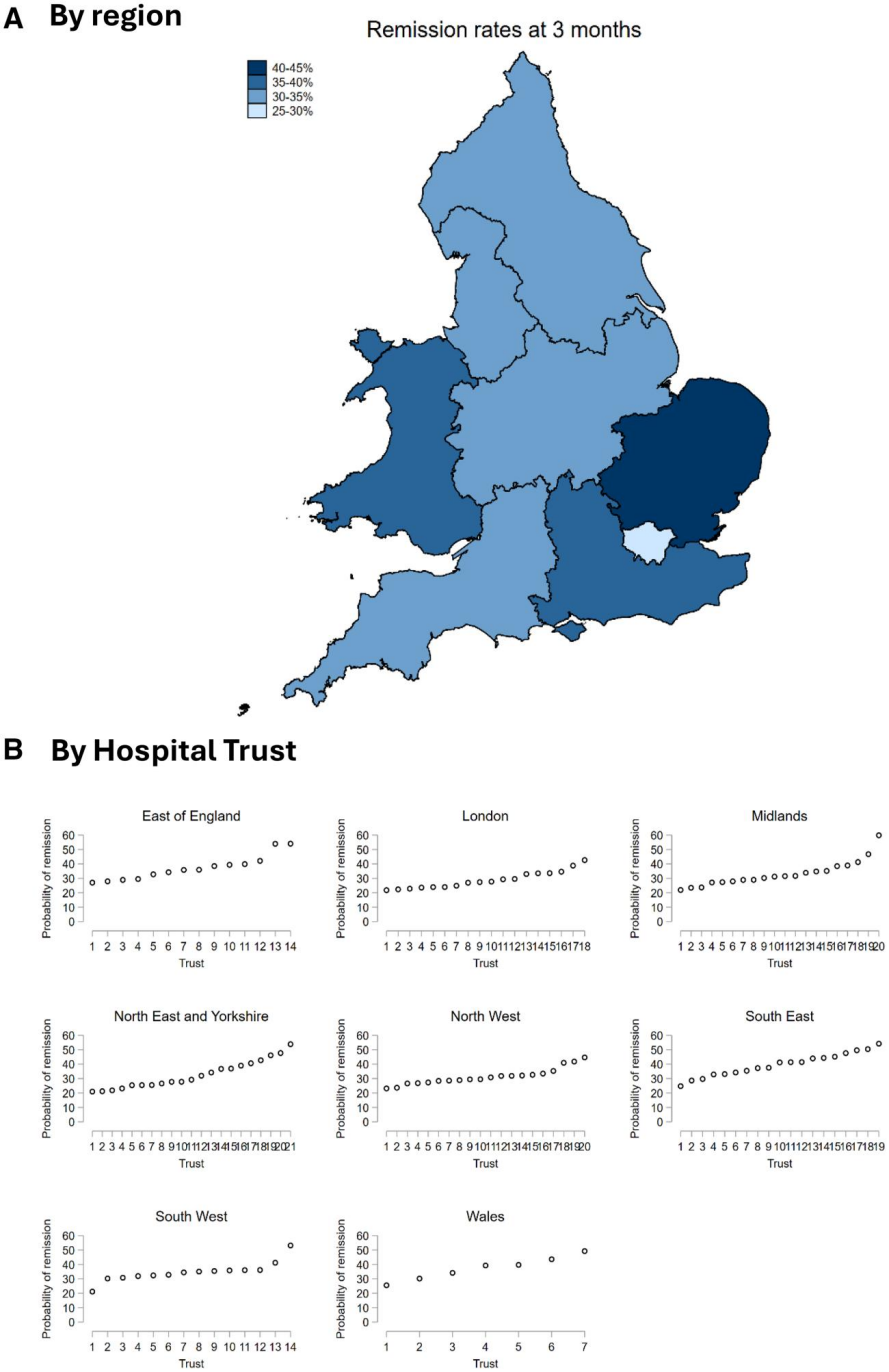

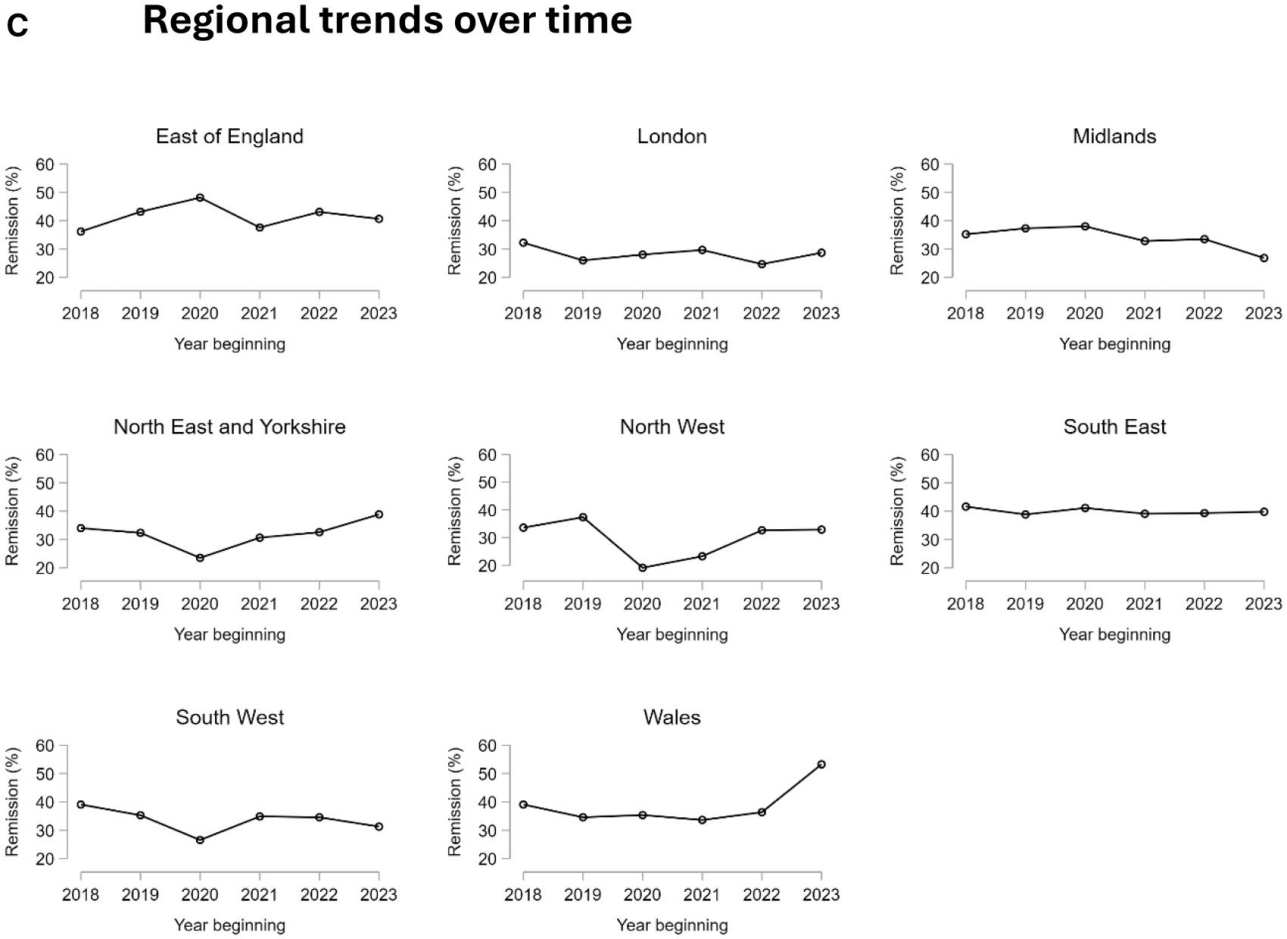
Probability of achieving remission (DAS28 < 2.6) at 3 months after initial rheumatology assessment for individuals with RA, shown by region of England and Wales (panel A), and Hospital Trust/Health Board within each region (panel B), alongside temporal trends in regional remission rates during the study period (panel C)

Underlying the relatively stable national trends in remission rates was marked variation between different regions and hospitals within England and Wales (Fig. 4; Supplementary Table S3, available at *Rheumatology* online). London had the lowest proportion of patients achieving remission at 3 months (28.4%), whilst the East of England (40.3%) and South East England (40.0%) had the highest remission rates (Fig. 4A; Supplementary Fig. S9 and Table S3, available at *Rheumatology* online). Within regions, variation in remission rates between individual rheumatology providers was even more marked, with patients at some Hospital Trusts being three times more likely to achieve remission than other Hospital Trusts within the same region (Fig. 4B). Temporal trends in remission rates also varied substantially by region over the study period (Fig. 4C), with improvements observed in some regions (e.g. Wales), while other regions saw worsening remission rates (e.g. Midlands).

## Discussion

Remission rates among people with RA in England and Wales have remained static since 2018, despite the implementation of a national audit programme aimed at driving down delays between presentation and treatment. After initial improvements in referral-to-treatment times, service delivery saw unprecedented disruption as a result of the COVID-19 pandemic and has still not recovered four years later. Beyond referral-to-treatment times, we demonstrated that numerous additional factors influence the probability of attaining early disease remission, particularly baseline disease severity and symptom duration prior to referral to rheumatology services. Importantly, a major impact of the pandemic has been increasing delays between symptom onset and presentation to primary care for individuals with RA, which need to be addressed if remission rates are to be improved going forwards.

Attainment of remission in RA is complex, and to understand the static remission rates observed at a national level, there are several factors that need to be considered in more detail. The pattern of static treatment responses held true when we assessed disease activity at 3 months according to DAS28 category (high vs. moderate vs. low disease activity vs. remission); when we assessed change in disease activity relative to baseline (EULAR treatment response); when we used different measures of remission attainment (DAS28-ESR vs. DAS28-CRP vs. Boolean remission) and when 12-month remission rates were analysed for those with available data. At an individual level, we demonstrated that the time from rheumatology referral to commencement of DMARDs is strongly predictive of remission, in keeping with findings from previous studies [11–13]. However, at a population level, we did not see improvement in remission rates—even before the pandemic, when referral-to-treatment times were improving—reflecting a disconnect between individual and population-level associations.

Contrasting these stable trends at a national level, we observed marked geographical variation in remission attainment by region and hospital. While improving remission rates were seen in some regions, others saw declining performance over the study period. Even greater variation was seen at the hospital level: remission attainment at individual hospitals within regions varied by up to a factor of three. This highlights the importance of capturing disaggregated hospital-level performance data through programmes such as NEIAA, which can then be used to target local quality improvement interventions.

We demonstrated that numerous factors beyond referral-to-treatment time are predictive of remission. These factors include patient characteristics, such as age, sex and ethnicity, and disease-related characteristics, such as baseline disease severity. Individuals with a baseline DAS28 of >5.1 were 60% less likely to achieve remission at 3 months than those with a DAS28 below this threshold. Importantly, we also demonstrated that symptom duration prior to referral was strongly predictive of remission, independently of the time taken to subsequently initiate DMARDs after referral. Moreover, we showed that the proportion of patients reporting symptom durations of >6 months has increased since the start of the pandemic. What is unclear from these data is what is driving this change. Patient behaviour may have altered, with individuals less likely to present early to primary care services operating at or beyond capacity, or having difficulty in obtaining appointments [14, 15]. These same service constraints may also have contributed to challenges in identifying patients with signs and symptoms of early inflammatory arthritis, at a time when there was a rapid transition to virtual consultations [16].

To contextualize the remission rates observed in this population-level study of early RA patients in England and Wales, we can compare these data with other study settings and healthcare systems. The 3-month DAS28 remission rates of 35% observed in our study are higher than those observed in the methotrexate-treated arms of contemporary RCTs that enrolled methotrexate-naïve participants with RA (e.g. 24% DAS28-CRP remission attainment at 24 weeks in RA Begin [17]; 18.5% DAS28-CRP remission at 24 weeks in ORAL Start [18] and 7.6% DAS28-ESR remission at 6 months in SELECT Early [19]). These differences might be due to the higher disease severity and longer disease duration at baseline of participants enrolled in these RCTs, relative to our real-world cohort. In cohorts of early RA patients initiating methotrexate in Sweden, Denmark and Norway, DAS28 remission attainment at 3 months ranged from 27% to 55% of participants [20]. There are likely to be numerous reasons for the widely-varying remission rates within these cohorts and ours, including differences in treatment protocols, visit frequency and concomitant corticosteroid usage (which ranged from 12% to 100% of participants) [20].

A major strength of our study was the ability to capture nationwide information at scale on both process measures and outcomes, with longitudinal data captured on over 20 000 individuals with RA from inception, making this one of the most comprehensive early RA cohorts globally. We used multiple models to explore predictors and patterns of remission, and we were able to account for numerous potential confounders of remission, including baseline disease severity.

There are also important limitations to our analyses. Foremost of these is that we have not been able to capture information on other factors that can contribute to remission attainment, including initial DMARD dose, adjuvant corticosteroid dose and duration, and granular data on treatment escalation regimens. Data on remission attainment at 3 months after initial rheumatology assessment are captured in NEIAA, with limited data on 12-month remission attainment for a minority of patients before April 2023; however, remission outcomes at other time points are not captured. As such, the extent to which treatment delays have a lasting impact on remission attainment cannot conclusively be determined from this study. Other key limitations include missing data on outcomes at follow-up, incomplete case ascertainment of new RA diagnoses (with the potential for preferential enrolment of patients and sampling bias) and the potential for unmeasured and/or residual confounding. One must also consider the methodological constraints during the COVID-19 pandemic, whereby the mandatory requirement for data collection in NEIAA was temporarily paused between March 2020 and May 2021; while this could be seen as a limitation (e.g. due to the potential for sampling bias), it also effectively represents a natural experiment that enabled us to explore the relationships between varying care delivery and clinical outcomes.

In conclusion, this study highlights that remission rates for individuals with RA have remained stable at a population level since the inception of NEIAA in 2018. However, underlying this national trend is wide variation in remission attainment at regional and hospital levels. We describe several factors that contribute to the likelihood of achieving remission for individuals newly diagnosed with RA, including symptom duration prior to referral, and referral-to-treatment time. Crucially, we have shown that people are now waiting longer to present to healthcare services in the post-pandemic era. Further work is needed to strengthen our understanding of the entire patient journey, from symptom onset through to treatment initiation and follow-up. NEIAA is a unique tool through which to provide insights into variation in rheumatology care, and highlight where improvements are most needed.

## Supplementary Material

keaf233_Supplementary_Data

## Data Availability

Data access requests can be made through the Healthcare Quality Improvement Partnership.

## References

[1] van Nies JAB , Tsonaka R, Gaujoux-Viala C, Fautrel B, van der Helm-van Mil AHM. Evaluating relationships between symptom duration and persistence of rheumatoid arthritis: does a window of opportunity exist? Results on the Leiden Early Arthritis Clinic and ESPOIR cohorts. Ann Rheum Dis 2015;74:806–12. 25561360 10.1136/annrheumdis-2014-206047

[2] Ostor AJ , Sawant R, Qi CZ et al Value of remission in patients with rheumatoid arthritis: a targeted review. Adv Ther 2022;39:75–93. 34787822 10.1007/s12325-021-01946-w PMC8799574

[3] NICE. Rheumatoid arthritis in adults: management. 2018. https://www.nice.org.uk/guidance/ng100/chapter/Recommendations.

[4] Smolen JS , Landewé RBM, Bergstra SA et al EULAR recommendations for the management of rheumatoid arthritis with synthetic and biological disease-modifying antirheumatic drugs: 2022 update. Ann Rheum Dis 2023;82:3–18. 36357155 10.1136/ard-2022-223356

[5] NICE. Rheumatoid arthritis in over 16s. 2020. https://www.nice.org.uk/guidance/qs33.

[6] British Society for Rheumatology. National Early Inflammatory Arthritis Audit. 2025.

[7] British Society for Rheumatology. National Early Inflammatory Arthritis Audit (NEIAA) State of the Nation Summary Report 2024. 2024. https://www.rheumatology.org.uk/improving-care/audits/neiaa.

[8] Studenic P , Aletaha D, de Wit M et al American College of Rheumatology/EULAR Remission Criteria for Rheumatoid Arthritis: 2022 revision. Arthritis Rheumatol 2023;75:15–22. 36274193 10.1002/art.42347 PMC10092655

[9] Fransen J , van Riel PL. The disease activity score and the EULAR response criteria. Rheum Dis Clin North Am 2009;35:745–57. vii–viii. 19962619 10.1016/j.rdc.2009.10.001

[10] Linden A. Conducting interrupted time-series analysis for single- and multiple-group comparisons. Stata J 2015;15:480–500.

[11] van der Heide A , Jacobs JW, Bijlsma JW et al The effectiveness of early treatment with "second-line" antirheumatic drugs. A randomized, controlled trial. Ann Intern Med 1996;124:699–707. 8633829 10.7326/0003-4819-124-8-199604150-00001

[12] Nell VP , Machold KP, Eberl G et al Benefit of very early referral and very early therapy with disease-modifying anti-rheumatic drugs in patients with early rheumatoid arthritis. Rheumatology (Oxford) 2004;43:906–14. 15113999 10.1093/rheumatology/keh199

[13] van der Linden MP , le Cessie S, Raza K et al Long-term impact of delay in assessment of patients with early arthritis. Arthritis Rheum 2010;62:3537–46. 20722031 10.1002/art.27692

[14] Royal College of General Practitioners. Key general practice statistics and insights. 2024. https://www.rcgp.org.uk/representing-you/key-statistics-insights#workload.

[15] British Medical Association. NHS backlog data analysis. 2024. https://www.bma.org.uk/advice-and-support/nhs-delivery-and-workforce/pressures/nhs-backlog-data-analysis.

[16] Green MA , McKee M, Katikireddi SV. Remote general practitioner consultations during COVID-19. Lancet Digital Health 2022;4:e7. 34952678 10.1016/S2589-7500(21)00279-X PMC8691847

[17] Fleischmann R , Schiff M, van der Heijde D et al Baricitinib, methotrexate, or combination in patients with rheumatoid arthritis and no or limited prior disease-modifying antirheumatic drug treatment. Arthritis Rheumatol 2017;69:506–17. 27723271 10.1002/art.39953 PMC5347954

[18] van Vollenhoven R , Takeuchi T, Pangan AL et al Efficacy and safety of upadacitinib monotherapy in methotrexate-naive patients with moderately-to-severely active rheumatoid arthritis (SELECT-EARLY): a multicenter, multi-country, randomized, double-blind, active comparator–controlled trial. Arthritis Rheumatol 2020;72:1607–20. 32638504 10.1002/art.41384 PMC7589375

[19] Lee EB , Fleischmann R, Hall S et al, ORAL Start Investigators. Tofacitinib versus methotrexate in rheumatoid arthritis. N Engl J Med 2014;370:2377–86. 24941177 10.1056/NEJMoa1310476

[20] Westerlind H , Glintborg B, Hammer HB et al Remission, response, retention and persistence to treatment with disease-modifying agents in patients with rheumatoid arthritis: a study of harmonised Swedish, Danish and Norwegian cohorts. RMD Open 2023;9:e003027. 37673441 10.1136/rmdopen-2023-003027 PMC10496677

