# Supplementary material for: Trends in remission rates for rheumatoid arthritis in England and Wales: a population-level cohort study
Source: Rheumatology (Oxford). 2025 May 5;64(9):4957–67. doi: 10.1093/rheumatology/keaf233 (PMC12407236; doi:10.1093/rheumatology/keaf233)
Supplement: keaf233_Supplementary_Data [file keaf233_supplementary_data.pdf]

Supplementary Figure S1. Disease activity (top panel) and EULAR treatment response states (bottom panel) at 3 months after initial rheumatology assessment for individuals with RA enrolled in NEIAA.

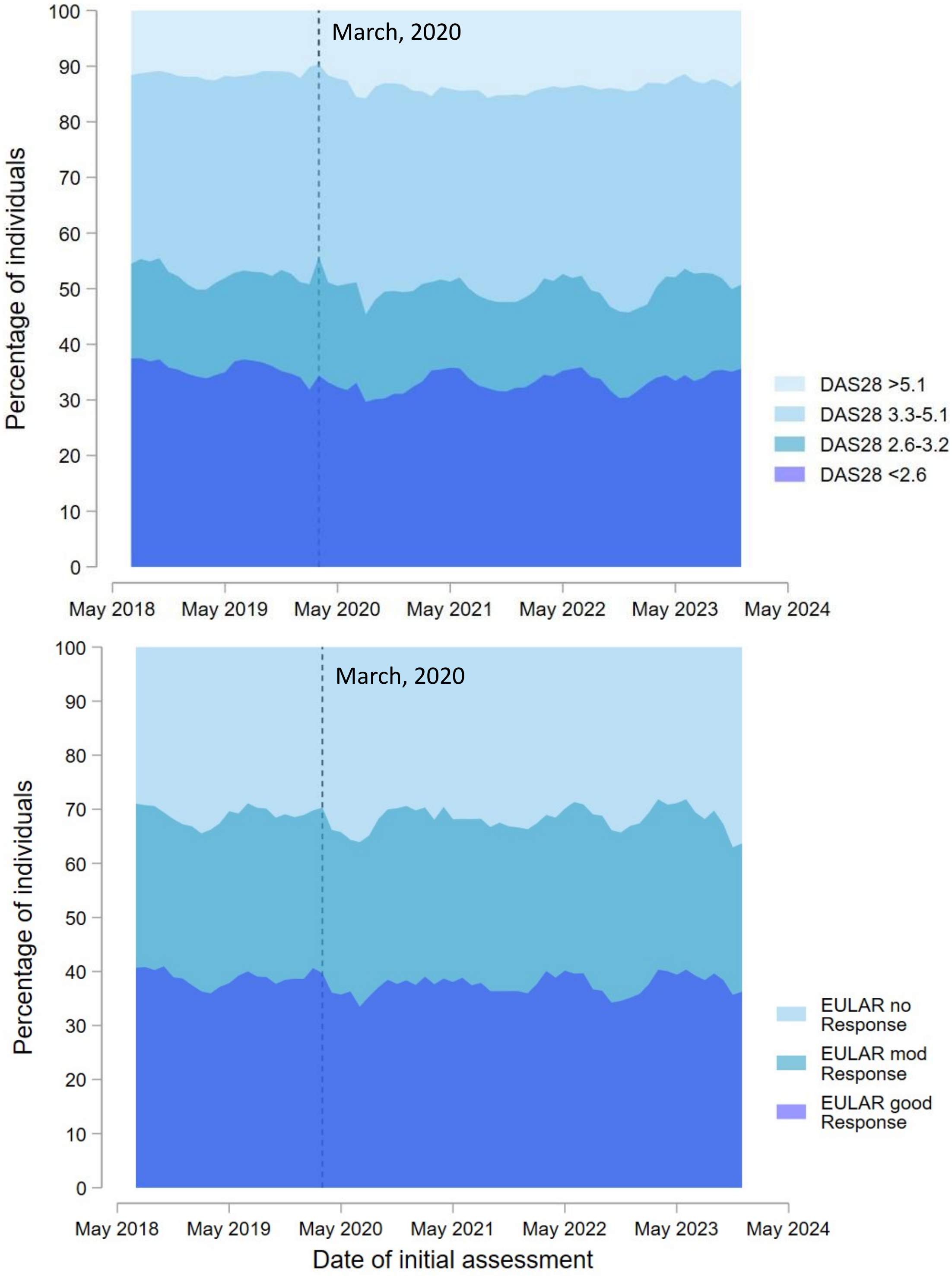

Smoothing has been applied, whereby each data point represents the average of the current month, the two preceding months, and the two subsequent months. The vertical dashed line corresponds to the onset of the first COVID-19 pandemic lockdown in England and Wales (March 2020). DAS28: Disease Activity Score at 3 months after initial assessment; EULAR response: EULAR treatment response at 3 months after initial assessment.

Supplementary Figure S2. Sensitivity analysis showing temporal trends in remission attainment at 3 months when using DAS28-CRP vs. DAS28-ESR vs. Boolean remission definitions.

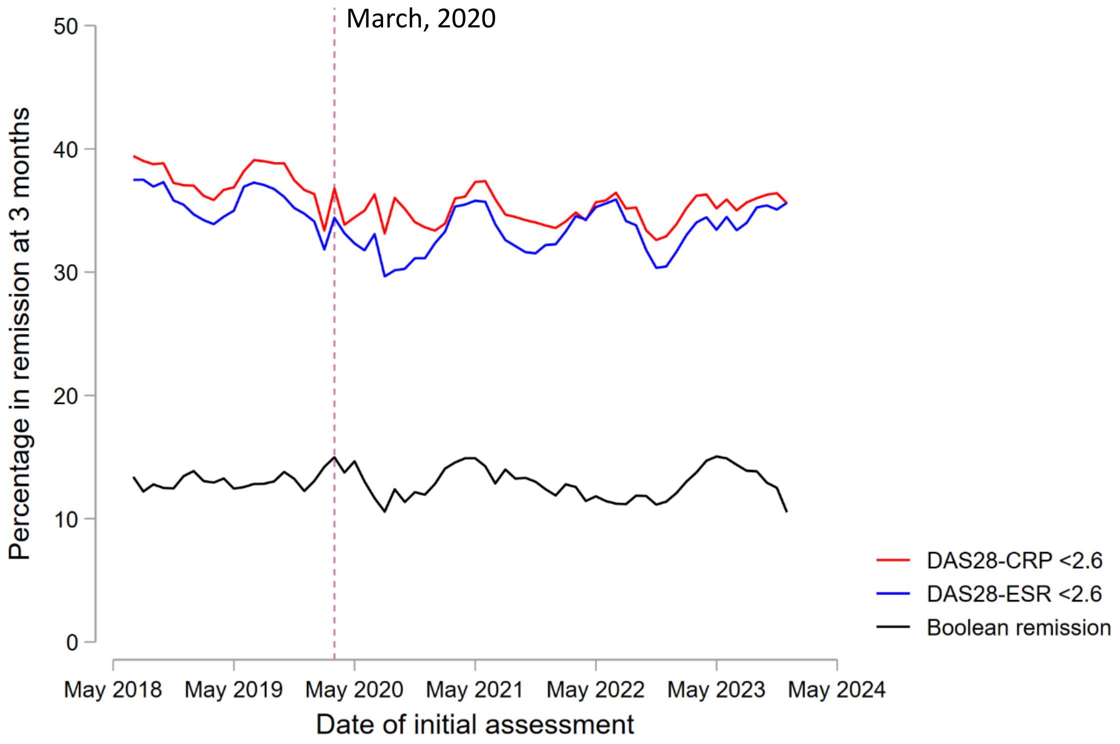

Temporal trends in the proportion of individuals with RA enrolled in NEIAA who achieved remission at the 3-month follow-up review. Separate lines are shown, utilising DAS28-CRP (*blue line*) or DAS28-ESR (*red line*) preferentially for individuals who had data available on both scores, as well as the Boolean definition of remission (*black line*). Smoothing has been applied, whereby each data point represents the average of the current month, the two preceding months, and the two subsequent months. The vertical dashed line corresponds to the onset of the first COVID-19 pandemic lockdown in England and Wales (March 2020).

Supplementary Figure S3. Temporal trends in remission attainment at 12 months after initial assessment for individuals with RA enrolled in NEIAA.

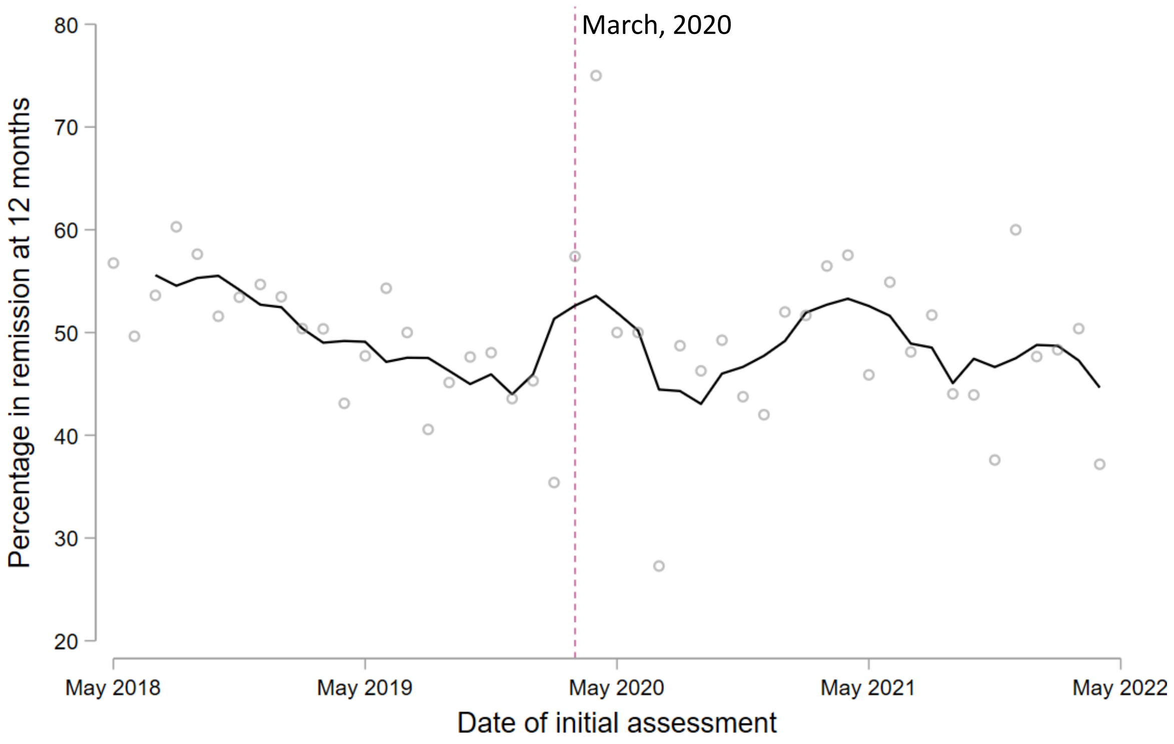

Data are shown for individuals with RA who had data available on DAS28 scores at 12 months after the initial rheumatology assessment. Individual data points are shown, in addition to a smoothed line that represents the average of the current month, the two preceding months, and the two subsequent months. The vertical dashed line corresponds to the onset of the first COVID-19 pandemic lockdown in England and Wales (March 2020).

Supplementary Figure S4. Interrupted time-series showing trends in the proportion of individuals with RA enrolled in NEIAA who received DMARDs within 6 weeks of being referred to rheumatology.

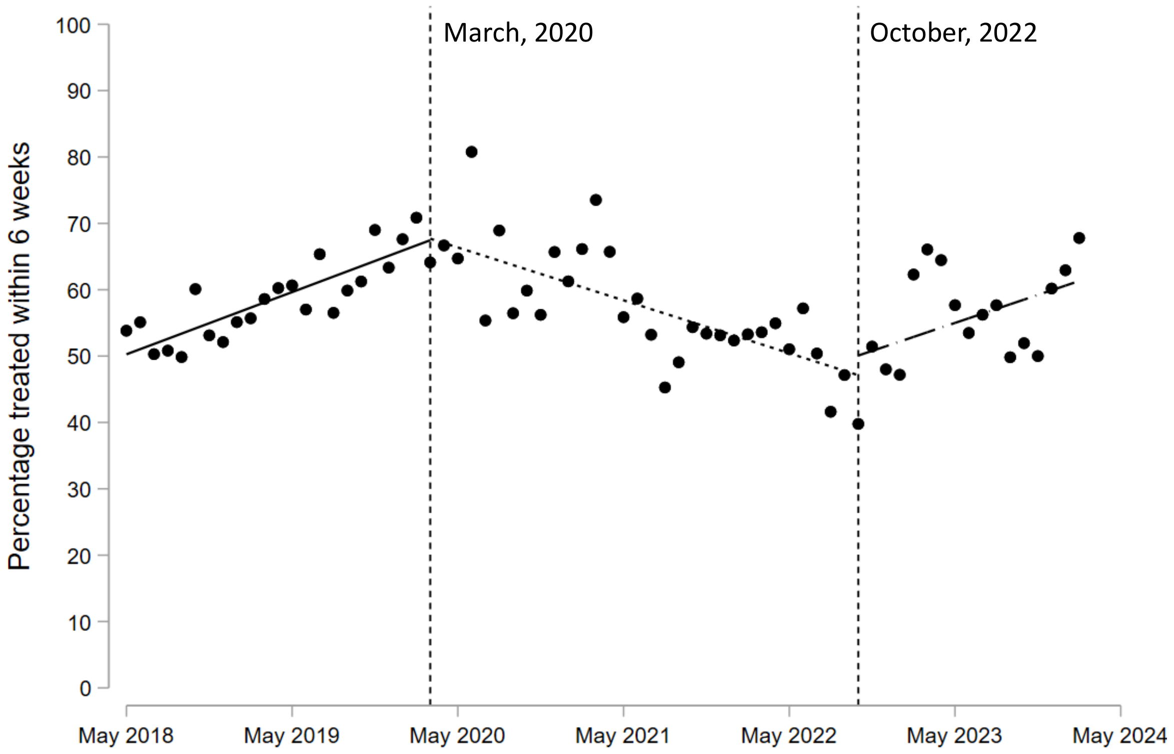

Individual data points represent the monthly averaged proportion of individuals with RA enrolled in NEIAA with were initiated on DMARDs within 6 weeks of referral to rheumatology services. The vertical dashed lines represent the start of the first COVID-19 lockdown in England and Wales (March 2020) and the nadir in post-pandemic referral-to-treatment performance (October 2022). The trend in performance prior to March 2020 was 9.38% improvement per year (95% CI 7.11 to 11.6; p<0.0001); the trend between March 2020 and October 2022 was 7.97% decrease per year (95% CI -6.38 to -9.55; p<0.0001); and the trend after October 2022 was 8.43% improvement per year (95% CI 0.71 to 16.1; p=0.032).

Supplementary Figure S5. Temporal trends in the proportion of individuals with RA enrolled in NEIAA who received DMARDs within 6 weeks (blue line) and within 12 weeks (red line) of referral to rheumatology services.

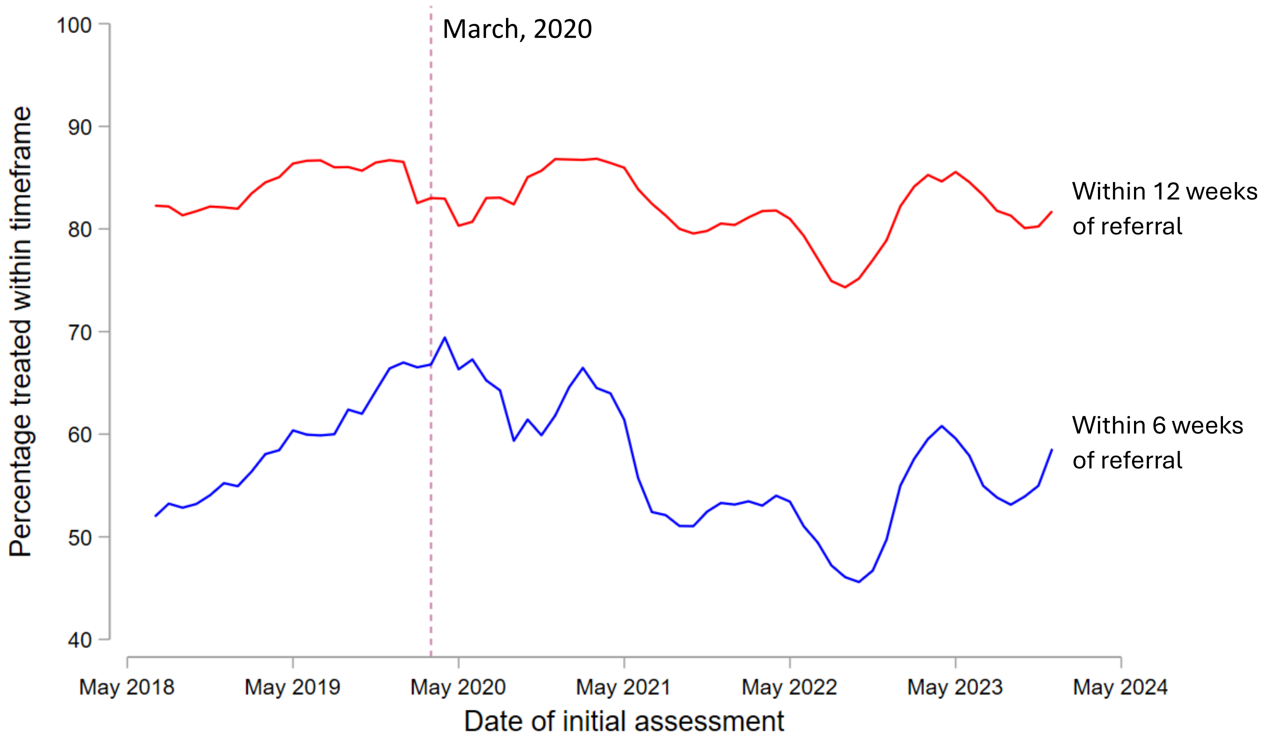

Smoothing has been applied, whereby each data point represents the average of the current month, the two preceding months, and the two subsequent months. The vertical dashed line corresponds to the onset of the first COVID-19 lockdown in England and Wales (March 2020).

Supplementary Figure S6. Interrupted time-series showing trends in the proportion of individuals with RA enrolled in NEIAA who received DMARDs within 12 weeks of referral to rheumatology.

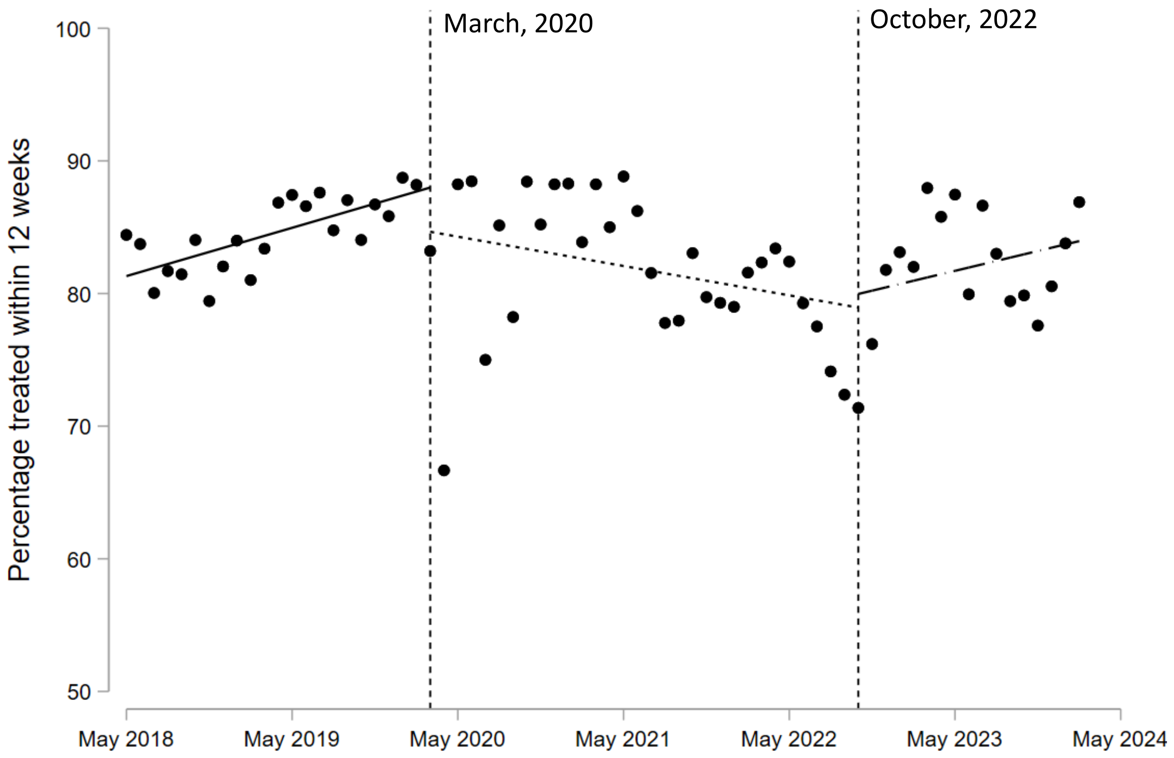

Individual data points represent the monthly averaged proportion of individuals with RA enrolled in NEIAA who were initiated on conventional synthetic DMARDs within 12 weeks of referral to rheumatology services. The vertical dashed lines represent the start of the first lockdown in England and Wales (March 2020) and the nadir in post-pandemic referral-to-treatment performance (October 2022). Trend prior to March 2020: 3.64% improvement per year (95% CI 2.26 to 5.02; p<0.0001); trend between March 2020 and October 2022: 2.21% decrease per year (95% CI -5.38 to 0.96; p=0.171); trend after October 2022: 2.99% improvement per year (95% CI -3.07 to 9.05; p=0.333).

Supplementary Figure S7. Interrupted time-series showing temporal trends in the proportion of patients with new diagnoses of RA reporting symptom durations of greater than 6 months prior to referral to rheumatology services.

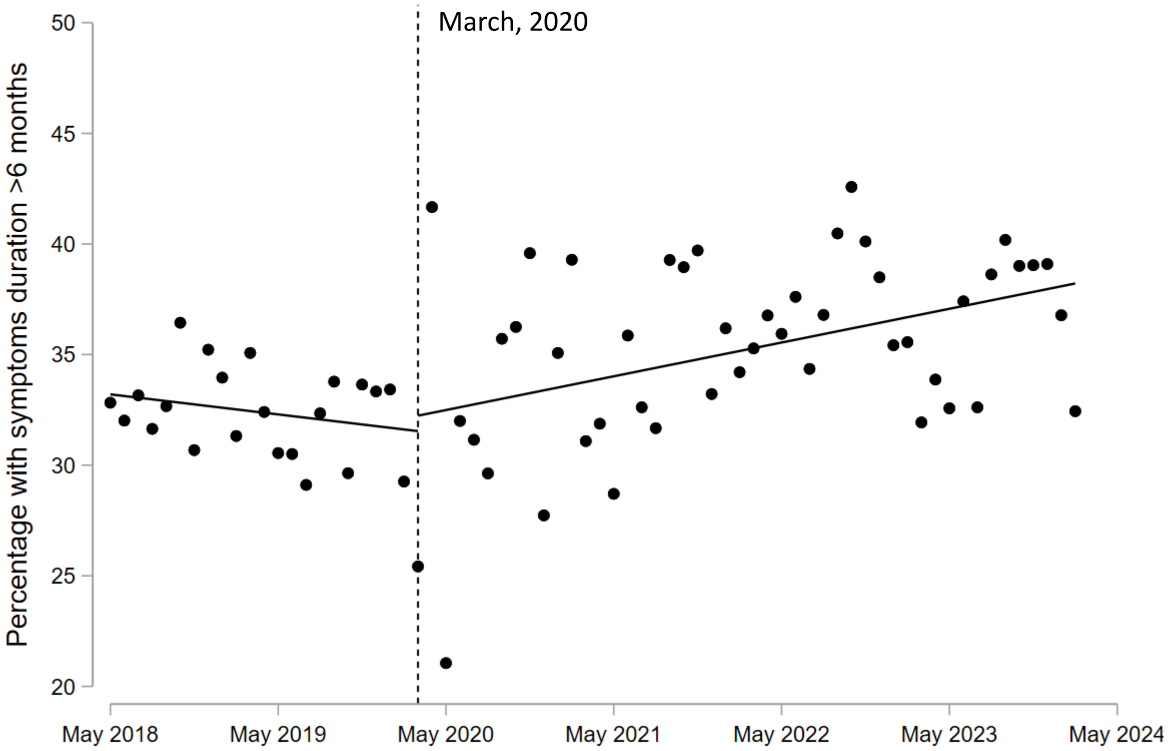

Individual data points represent the monthly averaged proportion of individuals with RA enrolled in NEIAA who had symptom durations of 6 months or more prior to referral to rheumatology services. The vertical dashed line represents the start of the first COVID-19 lockdown in England and Wales (March 2020). Trend prior to March 2020: 0.9% decrease per year (95% CI -1.86 to 0.0043; p=0.061); trend after March 2020: 1.52% increase per year (95% CI 0.55 to 2.50; p=0.002).

Supplementary Figure S8. Temporal trends in the proportion of individuals with RA enrolled in NEIAA who were referred to rheumatology services within 3 days of presenting to primary care with symptoms suggestive of early inflammatory arthritis.

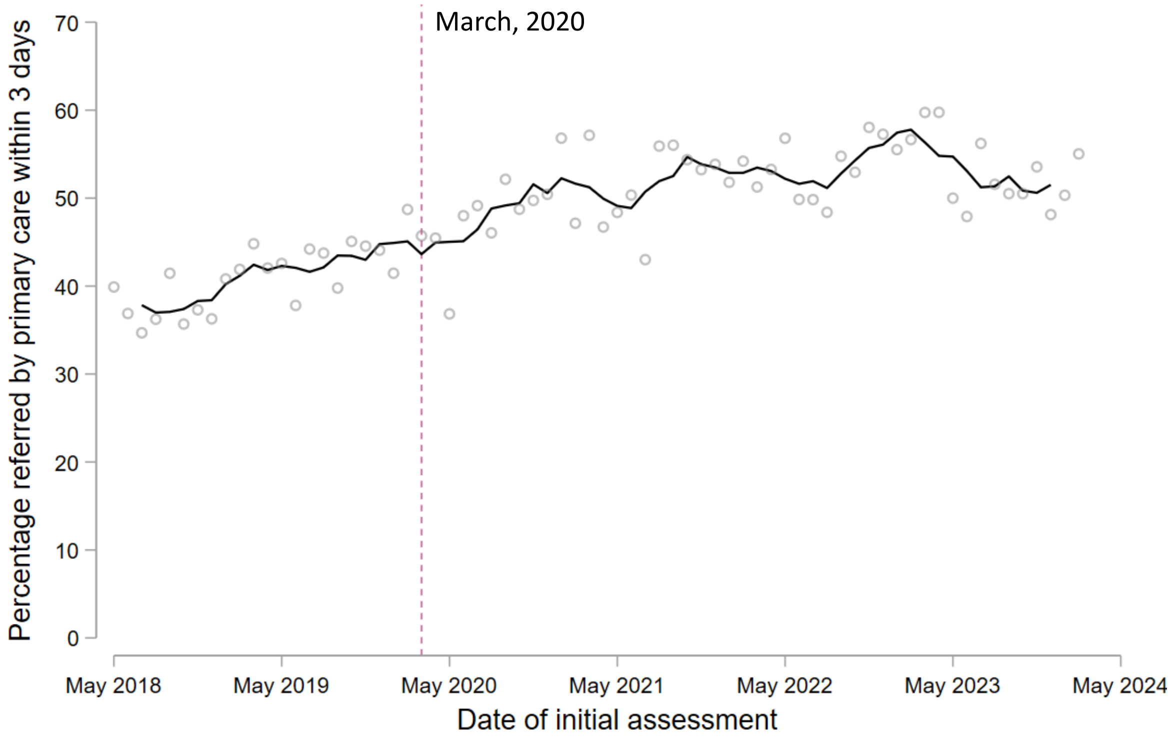

Individual data points are shown alongside a smoothed line that represents the average of the current month, the two preceding months, and the two subsequent months. The vertical dashed line corresponds to the onset of the first COVID-19 lockdown in England and Wales (March 2020).

Supplementary Figure S9. Regional variation in remission rates at 3 months for individuals with RA, shown with and without case-mix adjustment.

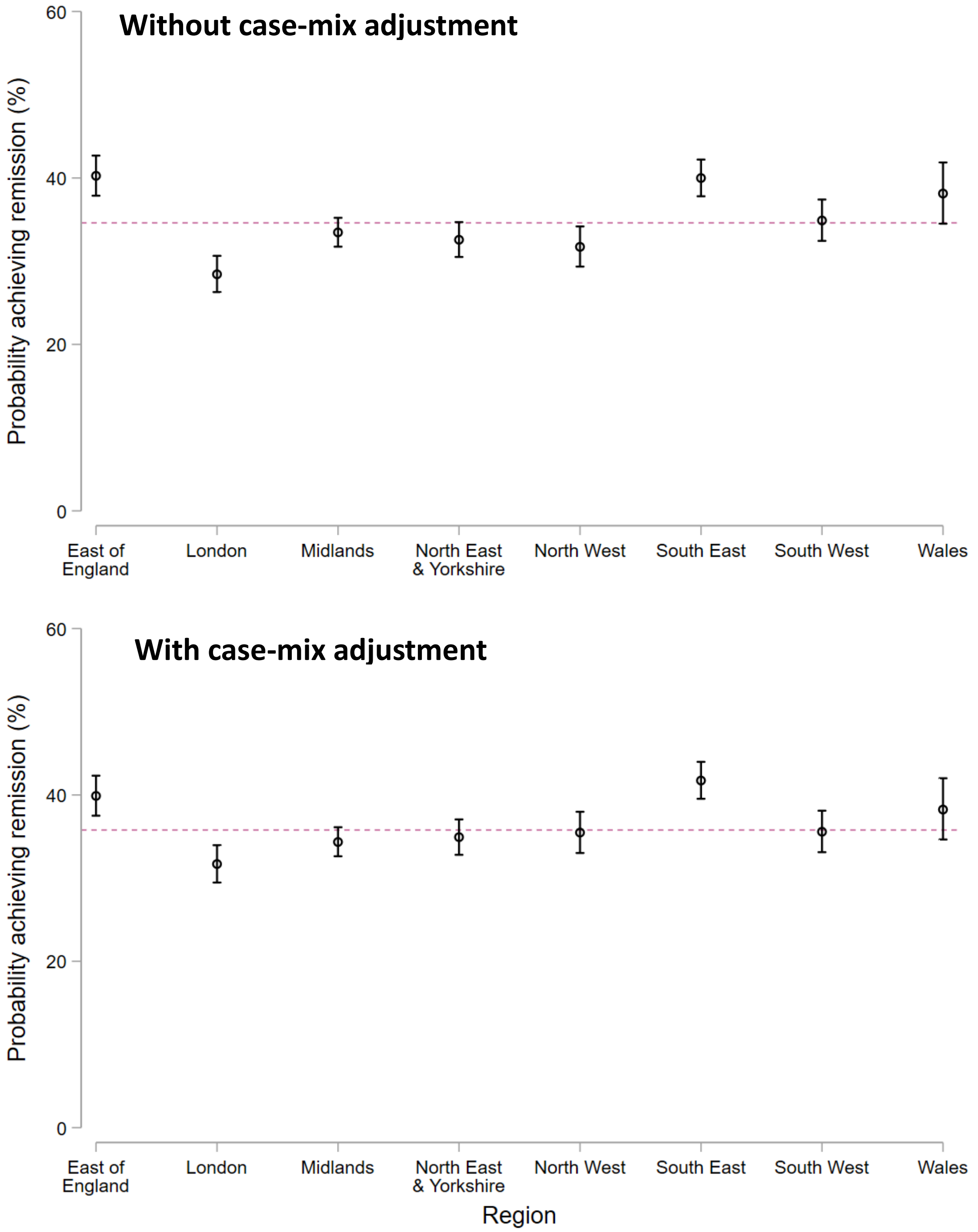

Estimates are shown by region of England and Wales, before (t*op panel*) and after (*bottom panel*) case-mix adjustment, with 95% confidence intervals. Case-mix adjustment was performed using the following variables held at constant levels (specified in brackets) across regions: age (50 years); sex (female); ethnicity (White); DAS28 at baseline (5.0). The horizontal dashed line in the top panel represents the observed proportion of individuals who achieved remission at 3 months, averaged across all regions. In the bottom panel, the horizontal dashed line represents the expected probability of remission following case-mix adjustment.

Supplementary Table S1. Predictors of achieving remission at 3 months after initial rheumatology assessment for individuals with RA in NEIAA.

		Univariable estimates	Age and sex-adjusted estimates	Multivariable-adjusted estimates
Age	Per 10-year increase	1.02 (0.99 to 1.05) p=0.23	1.01 (0.97 to 1.04) p=0.69	1.06 (1.02 to 1.09) p=0.0007
Sex	Male	Referent	Referent	Referent
	Female	0.77 (0.71 to 0.83) p<0.0001	0.77 (0.71 to 0.83) p<0.0001	0.79 (0.73 to 0.86) p<0.0001
Ethnicity	White	Referent	Referent	Referent
	Black	0.55 (0.42 to 0.71) p<0.0001	0.56 (0.43 to 0.73) p<0.0001	0.67 (0.50 to 0.90) p=0.0071
	Asian	0.88 (0.65 to 1.19) p=0.41	0.91 (0.67 to 1.23) p=0.53	0.91 (0.76 to 1.10) p=0.32
	Mixed/Other	1.02 (0.82 to 1.26) p=0.88	1.04 (0.84 to 1.29) p=0.73	1.00 (0.82 to 1.22) p=0.98
DAS28 at baseline	Per 1-unit increase	0.67 (0.64 to 0.69) p<0.0001	0.66 (0.64 to 0.68) p<0.0001	0.65 (0.63 to 0.67) p<0.0001
Autoantibody status	RhF and CCP negative	Referent	Referent	Referent
	RhF and/or CCP positive	1.05 (0.95 to 1.15) p=0.33	1.07 (0.98 to 1.17) p=0.13	0.99 (0.90 to 1.09) p=0.84
Time from referral to initiation of csDMARDs	0-3 weeks	Referent	Referent	Referent
	3-6 weeks	0.97 (0.86 to 1.09) p=0.57	0.97 (0.86 to 1.09) p=0.57	0.93 (0.82 to 1.05) p=0.26
	6-12 weeks	0.85 (0.74 to 0.97) p=0.017	0.85 (0.74 to 0.97) p=0.016	0.80 (0.69 to 0.92) p=0.0018
	>12 weeks	0.79 (0.66 to 0.94) p=0.0079	0.79 (0.66 to 0.94) p=0.0087	0.72 (0.60 to 0.86) p=0.0003
Duration of symptoms prior to referral	0-3 months	Referent	Referent	Referent
	3-6 months	0.92 (0.84 to 1.00) p=0.057	0.92 (0.85 to 1.01) p=0.073	0.92 (0.84 to 1.01) p=0.076
	6-12 months	0.82 (0.73 to 0.93) p=0.0026	0.83 (0.73 to 0.94) p=0.0031	0.81 (0.72 to 0.91) p=0.0004
	>1 year	0.77 (0.69 to 0.87) p<0.0001	0.78 (0.69 to 0.87) p<0.0001	0.72 (0.64 to 0.81) p<0.0001
Methotrexate commenced by 3 months	No	Referent	Referent	Referent
	Yes	0.91 (0.80 to 1.03) p=0.13	0.90 (0.79 to 1.02) p=0.11	1.09 (0.99 to 1.21) p=0.088
Corticosteroids prescribed at diagnosis	No	Referent	Referent	Referent
	Yes	0.77 (0.67 to 0.88) p=0.0001	0.76 (0.66 to 0.87) p=0.0001	1.05 (0.92 to 1.19) p=0.45

Estimates are from logistic regression models, reported as odds ratios with 95% confidence intervals. Univariable estimates are shown, along with age and sex-adjusted estimates, and estimates from a multivariable model including age, sex, ethnicity, DAS28 at baseline, autoantibody status, symptom duration prior to referral, time from referral to initiation of conventional synthetic DMARDs (csDMARDs), initiation of a methotrexate-based treatment regimen, and corticosteroid initiation at initial assessment. Multiple imputation was used for variables with missing data.

Supplementary Table S2. Sensitivity analysis using generalised ordinal logistic regression to explore associations between symptom duration, referral-to-treatment times, and the odds of achieving at least one category improvement in DAS28 score at 3 months after the initial rheumatology assessment.

		**Multivariable-adjusted estimates**
Duration of symptoms prior to referral	0-3 months	Referent
	3-6 months	0.93 (0.85 to 1.02) p=0.12
	6-12 months	0.82 (0.73 to 0.91) p=0.0002
	>1 year	0.73 (0.66 to 0.81) p<0.0001
Time from referral to initiation of csDMARDs	0-3 weeks	Referent
	3-6 weeks	0.94 (0.85 to 1.04) p=0.23
	6-12 weeks	0.81 (0.72 to 0.91) p=0.0003
	>12 weeks	0.71 (0.61 to 0.82) p<0.0001

Estimates comparing the odds of achieving a less active DAS28 category (high, moderate, low disease activity, or remission) than the current category, to those equally or more active than the current category. Proportional odds assumptions were met for symptom duration and the time from referral to initiation of conventional synthetic DMARDs (csDMARDs). All estimates were adjusted for age, sex, ethnicity, DAS28 at baseline, autoantibody status, initiation of a methotrexate-based treatment regimen by 3 months, and corticosteroid initiation at initial assessment.

Supplementary Table S3. Characteristics of individuals with RA diagnoses enrolled in NEIAA, separated by region of England and Wales.

	Total N=21,904	East of England N=2,580	London N=2,574	Midlands N=4,329	North East and Yorkshire N=3,100	North West N=2,525	South East N=3,081	South West N=2,573	Wales N=1,142
Mean age, years [SD]	59.4 (15.5)	60.5 (15.3)	55.1 (15.9)	59.1 (15.2)	59.5 (14.9)	59.6 (15.3)	59.5 (16.0)	61.9 (15.3)	60.2 (14.9)
Sex									
Male	7,977 (36.4%)	982 (38.1%)	802 (31.2%)	1,651 (38.1%)	1,123 (36.2%)	946 (37.5%)	1,104 (35.8%)	974 (37.9%)	395 (34.6%)
Female	13,927 (63.6%)	1,598 (61.9%)	1,772 (68.8%)	2,678 (61.9%)	1,977 (63.8%)	1,579 (62.5%)	1,977 (64.2%)	1,599 (62.1%)	747 (65.4%)
Ethnicity									
White	18,331 (84.8%)	2,358 (92.2%)	1,287 (50.0%)	3,579 (82.9%)	2,823 (91.2%)	2,289 (90.9%)	2,460 (85.5%)	2,446 (95.7%)	1,089 (96.3%)
Black	608 (2.8%)	43 (1.7%)	326 (12.7%)	98 (2.3%)	17 (0.5%)	37 (1.5%)	65 (2.3%)	20 (0.8%)	2 (0.2%)
Asian	1,788 (8.3%)	99 (3.9%)	637 (24.8%)	510 (11.8%)	133 (4.3%)	151 (6.0%)	183 (6.4%)	57 (2.2%)	18 (1.6%)
Mixed/Other	900 (4.2%)	58 (2.3%)	323 (12.6%)	132 (3.1%)	123 (4.0%)	42 (1.7%)	168 (5.8%)	32 (1.3%)	22 (1.9%)
Missing	277	22	1	10	4	6	205	18	11
RhF or CCP positive									
No	5,474 (26.5%)	708 (29.0%)	522 (21.5%)	936 (22.8%)	711 (23.7%)	683 (28.4%)	890 (33.5%)	768 (31.0%)	256 (22.8%)
Yes	15,155 (73.5%)	1,737 (71.0%)	1,908 (78.5%)	3,161 (77.2%)	2,287 (76.3%)	1,721 (71.6%)	1,768 (66.5%)	1,706 (69.0%)	867 (77.2%)
Missing	1,275	135	144	232	102	121	423	99	19
Median baseline DAS28 [IQR]	5.0 (4.0,5.9)	4.8 (3.8,5.9)	5.0 (4.0,5.9)	4.9 (3.8,5.9)	5.1 (4.1,6.0)	5.2 (4.2,6.1)	4.9 (3.8,5.9)	4.9 (4.0,5.8)	4.9 (4.0,5.9)
Missing	1,741	262	202	279	190	266	308	180	54
Duration of symptoms prior to referral									
<3 months	9,007 (41.4%)	1,087 (42.4%)	937 (36.5%)	1,776 (41.1%)	1,318 (43.2%)	1,041 (41.6%)	1,144 (37.5%)	1,204 (47.2%)	500 (43.9%)
3-6 months	5,227 (24.0%)	633 (24.7%)	625 (24.4%)	1,022 (23.7%)	742 (24.3%)	572 (22.9%)	806 (26.4%)	591 (23.1%)	236 (20.7%)
6-12 months	4,045 (18.6%)	473 (18.5%)	493 (19.2%)	795 (18.4%)	589 (19.3%)	490 (19.6%)	580 (19.0%)	420 (16.5%)	205 (18.0%)
>1 year	3,460 (15.9%)	370 (14.4%)	511 (19.9%)	724 (16.8%)	400 (13.1%)	398 (15.9%)	521 (17.1%)	338 (13.2%)	198 (17.4%)
Missing	165	17	8	12	51	24	30	20	3
Referral within 3 days of presentation									
No	11,166 (52.5%)	1,579 (65.3%)	1,285 (51.4%)	2,332 (54.5%)	1,482 (49.0%)	1,328 (54.9%)	1,306 (43.7%)	1,300 (51.8%)	554 (48.8%)
Yes	10,110 (47.5%)	838 (34.7%)	1,217 (48.6%)	1,943 (45.5%)	1,543 (51.0%)	1,091 (45.1%)	1,684 (56.3%)	1,212 (48.2%)	582 (51.2%)
Missing	628	163	72	54	75	106	91	61	6
Median time from referral to assessment, days [IQR]	17.0 (10.0,32.0)	19.0 (11.0,34.5)	18.0 (11.0,33.0)	18.0 (10.0,33.0)	15.0 (9.0,29.0)	17.0 (9.0,31.0)	19.0 (11.0,32.0)	16.0 (9.0,26.0)	21.0 (12.0,42.0)
Missing	162	8	35	37	18	11	23	29	1
Time from referral to first csDMARD									
Median time, days [IQR]	36.0 (20.0,66.0)	40.0 (21.0,70.0)	32.0 (19.0,62.0)	42.0 (21.5,76.0)	34.0 (19.0,62.0)	45.0 (25.0,77.0)	34.0 (18.0,62.0)	26.0 (15.0,46.0)	37.0 (21.0,75.0)
<3 weeks	5,017 (26.8%)	533 (23.6%)	625 (28.9%)	855 (23.5%)	733 (28.0%)	382 (18.3%)	773 (29.4%)	859 (37.5%)	257 (24.2%)
3-6 weeks	5,562 (29.7%)	646 (28.7%)	664 (30.7%)	964 (26.5%)	792 (30.3%)	577 (27.7%)	809 (30.8%)	777 (33.9%)	333 (31.4%)
6-12 weeks	4,927 (26.3%)	654 (29.0%)	542 (25.1%)	1,044 (28.6%)	714 (27.3%)	679 (32.6%)	607 (23.1%)	447 (19.5%)	240 (22.6%)
>12 weeks	3,237 (17.3%)	421 (18.7%)	332 (15.3%)	781 (21.4%)	378 (14.4%)	446 (21.4%)	440 (16.7%)	207 (9.0%)	232 (21.8%)
Missing	3,161	326	411	685	483	441	452	283	80
csDMARD commenced by 3 months									
No	2,780 (12.7%)	248 (9.6%)	374 (14.5%)	625 (14.4%)	452 (14.6%)	384 (15.2%)	399 (13.0%)	224 (8.7%)	74 (6.5%)

Yes	19,124 (87.3%)	2,332 (90.4%)	2,200 (85.5%)	3,704 (85.6%)	2,648 (85.4%)	2,141 (84.8%)	2,682 (87.0%)	2,349 (91.3%)	1,068 (93.5%)
Methotrexate commenced by 3 months									
No	8,373 (38.2%)	1,063 (41.2%)	1,245 (48.4%)	1,731 (40.0%)	1,254 (40.5%)	952 (37.7%)	1,067 (34.6%)	724 (28.1%)	337 (29.5%)
Yes	13,531 (61.8%)	1,517 (58.8%)	1,329 (51.6%)	2,598 (60.0%)	1,846 (59.5%)	1,573 (62.3%)	2,014 (65.4%)	1,849 (71.9%)	805 (70.5%)
Corticosteroids prescribed at diagnosis									
No	4,680 (21.8%)	549 (21.8%)	734 (29.8%)	814 (19.0%)	612 (20.0%)	419 (16.9%)	712 (23.6%)	530 (20.9%)	310 (27.6%)
Yes	16,808 (78.2%)	1,972 (78.2%)	1,733 (70.2%)	3,464 (81.0%)	2,446 (80.0%)	2,058 (83.1%)	2,311 (76.4%)	2,009 (79.1%)	815 (72.4%)
Missing	416	59	107	51	42	48	58	34	17
DAS28 <2.6 at 3 months									
No	8,988 (65.4%)	975 (59.7%)	1,211 (71.6%)	1,929 (66.5%)	1,331 (67.4%)	1,003 (68.3%)	1,163 (60.0%)	946 (65.1%)	430 (61.9%)
Yes	4,764 (34.6%)	657 (40.3%)	481 (28.4%)	970 (33.5%)	643 (32.6%)	466 (31.7%)	775 (40.0%)	507 (34.9%)	265 (38.1%)
Missing	8,152	948	882	1,430	1,126	1,056	1,143	1,120	447
Boolean remission at 3 months									
No	12,184 (87.1%)	1,419 (85.2%)	1,490 (88.2%)	2,538 (87.1%)	1,788 (87.9%)	1,357 (89.0%)	1,707 (85.4%)	1,276 (87.5%)	609 (86.1%)
Yes	1,807 (12.9%)	247 (14.8%)	199 (11.8%)	377 (12.9%)	245 (12.1%)	168 (11.0%)	291 (14.6%)	182 (12.5%)	98 (13.9%)
Missing	7,913	914	885	1,414	1,067	1,000	1,083	1,115	435
EULAR good response at 3 months									
No	8,225 (61.7%)	918 (57.7%)	1,108 (67.1%)	1,851 (65.4%)	1,197 (61.8%)	837 (60.9%)	1,085 (58.8%)	832 (58.8%)	397 (57.7%)
Yes	5,108 (38.3%)	674 (42.3%)	543 (32.9%)	979 (34.6%)	739 (38.2%)	538 (39.1%)	761 (41.2%)	583 (41.2%)	291 (42.3%)
Missing	8,571	988	923	1,499	1,164	1,150	1,235	1,158	454
Disease activity at 3 months									
DAS28 >5.1	1,759 (12.8%)	189 (11.6%)	259 (15.3%)	406 (14.0%)	247 (12.5%)	221 (15.0%)	199 (10.3%)	158 (10.9%)	80 (11.5%)
DAS28 3.3-5.1	4,928 (35.8%)	501 (30.7%)	642 (37.9%)	1,079 (37.2%)	759 (38.4%)	535 (36.4%)	648 (33.4%)	531 (36.5%)	233 (33.5%)
DAS28 2.6-3.2	2,301 (16.7%)	285 (17.5%)	310 (18.3%)	444 (15.3%)	325 (16.5%)	247 (16.8%)	316 (16.3%)	257 (17.7%)	117 (16.8%)
DAS28 <2.6	4,764 (34.6%)	657 (40.3%)	481 (28.4%)	970 (33.5%)	643 (32.6%)	466 (31.7%)	775 (40.0%)	507 (34.9%)	265 (38.1%)
Missing	8,152	882	1,430	1,126	1,056	1,143	1,120	447	882

Sociodemographic and disease characteristics are shown at baseline (the date of initial rheumatology assessment), in addition to treatment response metrics at 3 months following the initial rheumatology assessment. EULAR response represents the response to treatment (e.g. csDMARDs) at 3 months following initial assessment by a rheumatologist. csDMARD: conventional synthetic disease-modifying anti-rheumatic drug; RhF: rheumatoid factor; CCP: cyclic citrullinated peptide; DAS28: Disease Activity Score at 28 joints; SD: standard deviation; IQR: interquartile range.
